# MRI for Guided Right and Left Heart Cardiac Catheterization: A Prospective Study in Congenital Heart Disease

**DOI:** 10.1002/jmri.27426

**Published:** 2020-11-06

**Authors:** Mari Nieves Velasco Forte, Sébastien Roujol, Bram Ruijsink, Israel Valverde, Phuoc Duong, Nick Byrne, Sascha Krueger, Steffen Weiss, Yousef Arar, Surendranath R. Veeram Reddy, Tobias Schaeffter, Tarique Hussain, Reza Razavi, Kuberan Pushparajah

**Affiliations:** ^1^ School of Biomedical Engineering and Imaging Sciences King's College London London UK; ^2^ Department of Congenital Heart Disease Evelina London Children's Hospital, Guy's and St Thomas' NHS Foundation Trust London UK; ^3^ Cardiovascular Pathology Unit Institute of Biomedicine of Seville, IBIS, Virgen del Rocio University Hospital/CSIC/University of Seville Seville Spain; ^4^ Department of Medical Physics Guy's and St. Thomas' NHS Foundation Trust London UK; ^5^ Philips Research Europe Hamburg Germany; ^6^ Department of Pediatrics UT Southwestern Medical Center Dallas Texas USA

**Keywords:** congenital heart disease, cardiac catheterization, interventional MR, passive tracking

## Abstract

**Background:**

Improvements in outcomes for patients with congenital heart disease (CHD) have increased the need for diagnostic and interventional procedures. Cumulative radiation risk is a growing concern. MRI‐guided interventions are a promising ionizing radiation‐free, alternative approach.

**Purpose:**

To assess the feasibility of MRI‐guided catheterization in young patients with CHD using advanced visualization passive tracking techniques.

**Study Type:**

Prospective.

**Population:**

A total of 30 patients with CHD referred for MRI‐guided catheterization and pulmonary vascular resistance analysis (median age/weight: 4 years / 15 kg).

**Field Strength/Sequence:**

1.5T; partially saturated (pSAT) real‐time single‐shot balanced steady‐state free‐precession (bSSFP) sequence.

**Assessment:**

Images were visualized by a single viewer on the scanner console (interactive mode) or using a commercially available advanced visualization platform (iSuite, Philips). Image quality for anatomy and catheter visualization was evaluated by three cardiologists with >5 years' experience in MRI‐catheterization using a 1–5 scale (1, poor, 5, excellent). Catheter balloon signal‐to‐noise ratio (SNR), blood and myocardium SNR, catheter balloon/blood contrast‐to‐noise ratio (CNR), balloon/myocardium CNR, and blood/myocardium CNR were measured. Procedure findings, feasibility, and adverse events were recorded. A fraction of time in which the catheter was visible was compared between iSuite and the interactive mode.

**Statistical Tests:**

*T*‐test for numerical variables. Wilcoxon signed rank test for categorical variables.

**Results:**

Nine patients had right heart catheterization, 11 had both left and right heart catheterization, and 10 had single ventricle circulation. Nine patients underwent solely MRI‐guided catheterization. The mean score for anatomical visualization and contrast between balloon tip and soft tissue was 3.9 ± 0.9 and 4.5 ± 0.7, respectively. iSuite provided a significant improvement in the time during which the balloon was visible in relation to interactive imaging mode (66 ± 17% vs. 46 ± 14%, *P* < 0.05).

**Data Conclusion:**

MRI‐guided catheterizations were carried out safely and is feasible in children and adults with CHD. The pSAT sequence offered robust and simultaneous high contrast visualization of the catheter and cardiac anatomy.

**Level of Evidence:**

2

**Technical Efficacy Stage:**

1

CONGENITAL HEART DISEASE (CHD) is the most frequently occurring congenital disorder in newborns and the most frequent cause of infant death from birth defects.^1^ Improvements in diagnosis and treatment have led to a larger number of patients reaching adulthood, with an increase of 55% in the prevalence of adults living with severe types of CHD in the last decades.^2^ This rise in the population with CHD has made the secondary effects from current radiographic based techniques become more evident,^3^, ^4^ A recent study has shown a significant elevation in biomarkers of chromosomal damage both for diagnostic and therapeutic cardiac catheterization procedures, stating that no safe dose exists for radiation in children.^5^

Magnetic resonance imaging (MRI)‐guided catheterization offers an alternative to x‐ray‐guided cardiac procedures, avoiding radiation and providing better soft‐tissue visualization. In humans, passive tracking techniques have been used for diagnostic purposes in an increasing number of studies, with isolated series of successful interventions.^6^, ^7^, ^8^ Different visualization techniques involving passive tracking have been attempted using balloon wedge catheters. A carbon dioxide (CO_2_)‐filled balloon can be visualized as a signal void.^6^ Contrast‐based approaches use diluted gadolinium instead, which may be visualized by applying saturation prepulses intermittently,^9^ a black‐blood preparation using flow‐sensitive gradients,^10^ or with partial saturation (pSAT) prepulses.^11^ The latter option offers simultaneous visualization of the balloon, cardiac anatomy, and blood pool.

Cardiac procedures under MRI guidance in humans have been carried out using a single 2D viewer on the scanner console^6^, ^7^, ^8^ (referred to as interactive mode in this article) or by utilizing an advanced visualization platform in a separate workstation enabling real‐time visualization of 3D data and multiplane images, such as iSuite (Philips, Best, the Netherlands) or Interactive Front End (Siemens Corporate Research, Princeton, NJ).^12^, ^13^ The second option has been used for clinical purposes in electrophysiology (EP) studies and right heart catheterization for pulmonary vascular resistance analysis in adult and pediatric patients.^12^, ^13^, ^14^, ^15^, ^16^, ^17^

The purpose of this study was to evaluate the possibility of performing MRI‐guided catheterization and to assess the quality of visualization using a previously described pSAT sequence in an interactive mode or embedded in iSuite to optimize visualization for right and/or left heart catheterization in children (including infants) and young adults with CHD.

## Materials and Methods

Thirty‐four consecutive patients with CHD referred for pulmonary vascular resistance (PVR) analysis were considered for the study. The protocol was approved by the Institutional Review Board. Written informed consent was obtained from all participants or their legal representatives.

### In Vitro Validation

The entire setup was initially tested, optimized, and validated using a 3D‐printed heart phantom. The normal right heart of a 2‐year‐old patient was printed for this purpose.

The segmentation and standard tessellation language (STL) file preparation were performed manually using Mimics software (v. 18.0, Materialise, Leuven, Belgium) and 3‐Matic Medical (v. 10.0, Materialise). The model was printed using polyjet technology in Tango Plus FullCure930 material in a hollow fashion to allow the simulation of the intervention. The inferior vena cava (IVC) and superior vena cava (SVC) were artificially elongated in order to facilitate the phantom experiments. The 3D model was then attached to a water‐filled plastic box using six French introducers connected at each end.

Visualization and navigation were tested using a previously described pSAT sequence^11^ in interactive mode or embedded in iSuite. A 5Fr balloon wedge catheter was used, inflated with 1–2 mL of a 1% dilution of gadolinium (Dotarem) at the balloon tip for all experiments.

**FIGURE 1:**
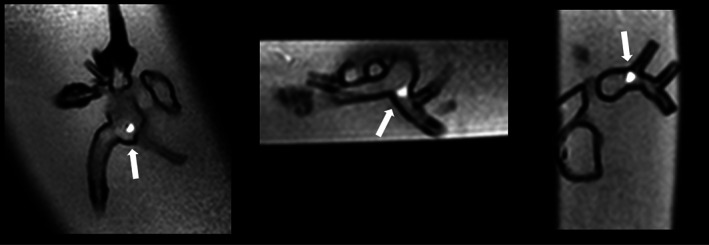
Visualization of the balloon wedge catheter in a 3D printed heart. Tip of the catheter at the right atrium (left), origin of the left pulmonary artery (center) and in the right pulmonary artery (right).

Each operator performed the procedure three times. Catheterization was performed following the same strategy and route as in clinical cases. First, a 3D bSSFP was acquired and selected imaging planes were stored when iSuite was utilized. The catheter was then navigated from the IVC to the right atrium, right ventricle, main pulmonary artery (PA), and PA branches (Fig. 1). A 5‐channel cardiac array receiver coil was used for the experiments.

### *Real‐Time* *MRI‐Guided Cardiac Catheterization*

Imaging was performed on a Philips XMR setup (Achieva or Ingenia, Philips, Best, Netherlands). This consists of a 1.5T MRI‐scanner and a BV Pulsera or Allura Clarity or Ingenia (Philips) cardiac X‐Ray unit. All studies were conducted under general anesthesia based on clinical need. An appropriate size balloon wedge catheter (Arrow Intl., Reading, PA) and receiver coil were used depending on the weight of the patient (Table 1).

**TABLE 1:** Diagnosis, Previous Procedures, Weight, Slice Thickness, pSAT Angle, Size of Balloon Wedge Catheter, Coil, and Scanning Method Used for Each Patient

Patient	Diagnosis	Previous cardiac procedures	Weight (kg)	Slice thickness/pSAT flip angle	Heart structures catheterized	Size of balloon wedge catheter (French)/Use of MRI compatible wire	Coil	Scanning method
1	Complete AVSD	Nil	49	10 mm/30°	IVC, RA, RV, MPA, LPA, LCPW	6/No	Cardiac coil	Interactive
2^a^	Allagile's syndrome. Bilateral PAs stenosis	Nil	7.5	10 mm/30°	IVC, RA, RV, MPA, LPA, RPA, RPCW, DAo, AAo, LV	4/No	Cardiac coil	iSuite
3	DORV, malposition of the great arteries William's Syndrome Asthma Upper airway obstruction (partial vocal palsy)	• Modified BT shunt • VSD closure, ASO (Lecompte), excision of subpulmonary stenosis	98	8 mm/50°	IVC, RA, RV, RPA, MPA, DAo, AAo, LV	6/No	Cardiac coil	iSuite
4^a^	Allagile, PAs stenosis	Nil	12.2	5 mm/30°	IVC, RA, RV, MPA, LPA, RPA, RPCW, DAo, AAo, LV	4/No	Cardiac coil	iSuite
5	Left atrial isomerism Interrupted IVC with bilateral azygos continuation PAPVD (RPV to right sided atrium), small ASD, perimembranous VSD Pulmonary hypertension	• Complete surgical repair	14	7 mm/30°	IVC, RA, RV, MPA, LPA, RPA, RPCW, DAo, AAo, LV	4/No	Cardiac coil	Interactive
6	TGA, AP window	Nil	11	7 mm/30°	IVC, RA, RV, MPA, LPA, LPCW, Aorta	4 (v) & 5 (a)/No	Cardiac coil	iSuite
7	AVSD	Nil	6.5	7 mm/40°	IVC, LA, RA, RV, LV, MPA, LPA, RPA, RCPW	4/No	Flex M	iSuite
8	TOF	• Balloon dilatation of pulmonary valve • Surgical repair of TOF & LPA reconstruction • Balloon dilatation of right upper, RUPA, RMPA &RLPA • Balloon dilatation of LPA & stent insertion	33	10 mm/30°‐45°	IVC, RA, RV, MPA, RPA, (LPA attempted but obstructed), RCPW	6/No	Cardiac coil	iSuite
9	HLHS	Single ventricle – s/p Fontan	45	8 mm/40°	IVC, LL, SVC, RPA, LPA, LPCW, RA, LA, RV	6/No	Cardiac coil	iSuite
10	TAPVD, severe RPV stenosis	• Surgical repair of TAPVD • Balloon dilatation of RUPV	25	7 mm/40°	IVC, RA, RV, LPA, RPA, RPCW, LPCW, DAo, AAo, LV	5 (v) & 4 (a)/No	Cardiac coil	iSuite
11	Secundum ASD, Perimembranous VSD, T21	Nil	6	7 mm/40°	IVC, RA, LA, RV, MPA, LPA, RPA, LPCW,	4/No	Flex L	iSuite
12	PAPVD (LU&LMPV to innominate vein), perimbranous VSD	Nil	5	7 mm/40°	IVC, RA, RV, LPA, RPA, LPCW, RPCW. DAo, AAo, LV	4/No	Flex L	iSuite
13	AV discordance, DORV	• Single ventricle – s/p Fontan	60	8 mm/30°	IVC, LL, SVC, RA, LA, RV, RPA, LPA, LPCW	6/No	Cardiac coil	iSuite
14	PAPVD (RPV to RA), secundum ASD	• Coil embolisation of MAPCAs	5.5	N/A	IVC, RA, RV, MPA, LPA (RPA attempted but obstructed), LA, LV, DAo, Asc Ao	4/No	Cardiac coil	N/A
15	HLHS	Single ventricle – s/p Fontan	20	7 mm/30°	IVC, LL, SVC, RPA, LPA, LPCW, RA, LA, RV	6/No	Cardiac coil	iSuite
16	Criss‐cross heart, ASD, severe subpulmonary and pulmonary valve stenosis	Nil	19	N/A	IVC, RA, RV, MPA, RPA, LPA, RPCW	5/No	Cardiac coil	N/A
17	Discordant AV connections, concordant VA connections	• Single ventricle – s/p Glenn	11.5	8 mm/30°	IVC, RA, LV, SVC, RPA, LPA, LPCW	4/No	Torso coil	Interactive
18	HLHS	• Single ventricle – s/p Fontan	21.3	8 mm/40°	IVC, LL, SVC, RPA, LPA, LPCW, RA, LA, RV	6/No	Torso coil	Interactive
19	HLHS	• Single ventricle – s/p Glenn	18	8 mm/40°	IVC, RA, RV, SVC, RPA, LPA, LPCW	5/No	Torso coil	Interactive
20	Aortic Coarctation, moderate sized VSD, congenital left diaphragmatic hernia and left lung hypoplasia, 13 chromosome deletion, scoliosis	• Aortic coarctation repair	7	10 mm/40°	IVC, RA, RV, MPA, RPA, RPCW, LPA, LPCW, LV, AAo, DAo	4/Yes	Cardiac coil	iSuite
21	Common atrium, tiny muscular VSD	• ASD closure	34	10 mm/40°	IVC, RA, RV, MPA, RPA, LPA, LPWC	6/Yes	Cardiac coil	iSuite
22	HLHS, Left pulmonary vein stenosis	• Single ventricle – s/p HemiFontan • LPV stent	13	N/A	SVC, LPA, LPCW, RPA, RPCW	4/No	Cardiac coil	N/A
23	Perimembranous ventricular septal defect	Nil	15	10 and 5 mm/40°	IVC, RA, RV, MPA, LPA, RPA, RPCW, LV, AAo, DAo	5/No	Cardiac coil	iSuite
24	HLHS	• Single ventricle – s/p Fontan • LPA stent	24	N/A	IVC, LL, LPA, RPA, RPCW, RA, RV, AAo, DAo	5 (v) & 4 (a)/No	Cardiac coil	N/A
25^a^	HLHS	• Single ventricle palliation – s/p Fontan	72	10 mm/40°	IVC, LL, SVC, RPA, LPA, LPCW, RA, RV, AAo, DAo	6Fr (v & a)/Yes	Cardiac coil	iSuite
26	Heterotaxy, dextrocardia, AVSD, sup/inf ventricles, pulmonary atresia with disconnected PAs	• Unifocalization of MAPCAs + BTS	7.6	8 mm/30°	IVC, RA, LA, RV, LV, DAo, AAo, BTS, LPA, RPA	5 (v) & 4 (a)/No	Flex M	Interactive
27	TOF/absent pulmonary valve	• Ao homograft, LeCompte maneuver	13.3	10 mm/40°	IVC, RA, RV, MPA, LPA, RPA, RCPW	5/No	Flex M	Interactive
28	TGA, hypoplastic RV	• Single ventricle – s/p Fontan	17.4	10 mm/40°	IVC, LL, SVC, LPA, RPA	6/No	Flex M	Interactive
29	HLHS	• Single ventricle – s/p Glenn	13.4	8 mm/40°	IVC, RA, RV, SVC, RPA, LPA, LPCW	5 (v) & 4 (a)/No	Flex M	Interactive
30	HLHS	• Single ventricle – s/p Glenn	15.4	8 mm/40°	IVC, RA, RV, SVC, RPA, LPA, LPCW	5 (v) & 4 (a)/No	Flex M	Interactive

The size of the sheath refers to the venous access unless specified otherwise.

^a^ These patients underwent dobutamine stress study as well as PVR analysis.

(A) = arterial access; ASD = atrial septal defect; AAo = ascending aorta; AV = atrioventricular; AVSD = atrioventricular septal defect; BTS = Blalock‐Taussig shunt; DAo = descending aorta; DORV = double outlet right ventricle; HLHS = hypoplastic left heart syndrome; IVC = inferior vena cava; LA = left atrium; LL = lateral tunnel; LPA = left pulmonary artery; LPCW = left pulmonary capillary wedge; LPV = left pulmonary veins; LV = left ventricle; MAPCA = major aortopulmonary collateral artery; PAs = pulmonary arteries; RA = right atrium; RPA = right pulmonary artery; RPCW = right pulmonary capillary wedge; RUPA = right upper pulmonary artery; RMPA = right middle pulmonary artery; RLPA = right lower pulmonary artery; RV = right ventricle; RVOT = right ventricular outflow tract; SVC = superior vena cava; TGA = transposition of the great arteries; TOF = Tetralogy of Fallot; (v) = venous access; VSD = ventricular septal defect.

Access to the right or left femoral vein and artery was gained in the x‐ray part of the XMR suite. Once access was gained, the patient was transferred to the scanner and an appropriate size catheter was inserted. The balloon at the catheter tip was filled in with a dilution of 1% gadolinium (Dotarem, Guerbet, France) mixed with a physiological 0.9% sodium chloride solution. Balloons were initially inflated to a maximal volume as recommended by the manufacturer. However, the volume injected in the balloon‐tip was adapted to the size of the navigated structures. This was particularly necessary when approaching the PA branches, where the size of the vessels reduced in caliber in relation to the diameter of the feeding vessel, and where stenosis is a common finding in patients with CHD.

The iSuite platform consists of a PC located next to the scanner console where the software is installed, two mirrored screens (one next to the iSuite PC in the scanner console room and one in the scanner room), and a set of foot pedals. The scanner console and the iSuite PC were electronically connected and images sent automatically in real‐time. For each case, after survey and localizers scans, a 3D balanced steady‐state free‐precession (bSSFP) sequence was acquired at the beginning of the study. Using the 3D bSSFP images imported into iSuite, patient‐specific imaging planes were defined for the following anatomical views: bicaval (multiplanar reformat showing the SVC and IVC), 4‐chamber, right ventricular outflow tract (RVOT), right 3‐chamber (R3ch), pulmonary artery (PA) bifurcation, right and left pulmonary arteries (RPA and LPA, respectively) for right heart catheterization. Left ventricular outflow tract (LVOT), left 3‐chamber (L3ch), and aortic arch anatomies were also stored for patients needing left heart investigations. Pressures were measured and recorded across all cardiac chambers catheterized. In three cases an MRI conditional wire (EP flex Feinwerktechnik, Germany, 0.035″ diameter and 180 cm length) was used.

The iSuite screen in the scanner room was used for the operator's guidance. Real‐time imaging was controlled by the foot pedals. These could be used to switch between a standard single‐plane acquisition and dynamic interleaved acquisitions of two or three orthogonal imaging planes that could be displayed simultaneously, and to start or stop imaging. The operator was able to navigate backwards and forwards in the same plane orientation by pressing the pedals, each movement advanced 80% of the previously defined slice thickness of the sequence. For catheterization guidance, a previously described pSAT sequence (repetition time [TR] / echo time [TE] = 2.65 msec/1.3 msec, flip angle = 60°, field of view [FOV] = 350 × 300 mm^2^, voxel size = 2.2 × 2.5 mm^2^, slice thickness = 10 mm, bandwidth = 1250 Hz/pixel, SENSE factor = 2, partial *k*‐space acquisition in the phase‐encoding direction = 65%, number of phase encoding lines = 40, acquisition time = 106 msec) was used.^11^ The pSAT angle to obtain partial saturation was optimized for visualization in each case. The slice thickness was adapted depending on the patient's weight and the size of the structures that needed to be catheterized (Table 1).

### *Image Quality Assessment and Feasibility of* *MRI* *Catheterization*

Image quality for anatomy and catheter visualization was retrospectively evaluated by three cardiologists (I.V., T.H., K.P.) with 11, 12, and 10 years of experience in cross‐sectional imaging and MRI catheterization, respectively, using a 1–5 scale (1, poor, 5, excellent).^11^

Catheter balloon signal‐to‐noise ratio (SNR), blood SNR, myocardium SNR, catheter balloon/blood contrast‐to‐noise ratio (CNR), catheter balloon/myocardium CNR, and blood/myocardium CNR were measured for each case, as previously described.^11^

Demographic data, diagnosis, mean heart rate and end‐tidal CO_2_ (etCO_2_) during the procedure, radiation dose and time, pulmonary vascular resistance at rest and under inhaled vasodilators, and adverse events were recorded. As per institutional protocol, baseline PVR was assessed under 30% fraction of inspired oxygen (FiO_2_) and repeated with inhaled pulmonary vasodilators and 100% fraction of inspired oxygen when indicated.

For each patient, we evaluated the feasibility of MRI‐guided catheterization and fraction of time during MRI catheterization in which the catheter was visible in at least one plane of space. Slice thickness and the pSAT angle used during catheterization were recorded. All medical personnel involved in MRI‐guided interventions at our institutions were trained in MRI safety before participation in clinical scenarios.

### Statistical Analysis

Statistical analysis was performed with IBM SPSS v. 20.0 (Armonk, NY). Numerical values are presented as mean ± standard deviation (SD) unless stated otherwise. The fraction of time in which the catheter was visible was compared between iSuite and interactive mode. A *t*‐test was used to test for differences between the two guidance approaches in terms of fraction of time in which the catheter was visible. For categorical variables (scores), a Wilcoxon signed rank test was performed to evaluate statistical significance. Categorical variables are presented as median and range unless stated otherwise. *P* < 0.05 was considered significant.

## Results

### Demographic Features and Procedure Findings

From the 34 consecutive patients initially referred for PVR study, 30 patients were finally recruited (two patients declined enrolment, one patient had a pacemaker implanted, and one patient was an adult without capacity to consent).

Demographic features and hemodynamic findings are summarized in Table 2. Among all recruited patients, 11 were females. The median age was 4 years (range: 2 months to 39 years), median weight was 15.2 kg (range: 5–98 kg), median body surface area (BSA) was 0.65 m^2^ (range: 0.27–2). Twenty patients had previous major cardiac surgery. Three needed a dobutamine stress study in addition to baseline PVR analysis. Seven underwent right heart catheterization, 11 needed both right and left heart assessment, and the rest of the patients^12^ were single ventricle anatomies needing catheterization of the Fontan (seven cases) or HemiFontan/Glenn (five cases) circulation and intracardiac pressures.

**TABLE 2:** Summary of Demographic Features and Catheterization Details in Recruited Patients

Age (median, range)	4 years (2 months to 39 years)
Body surface area (median, range)	0.65 m^2^ (0.27–2)
Weight (median, range)	15.2 kg (5–98)
Height (median, range)	102 cm (58–175)
Previous cardiac procedure	20/30 patients
Heart rate	98 ± 28 bpm
Catheterisation time (skin in‐skin out)	219 ± 52 minutes
Indexed radiation dose	0.42 ± 0.58 mGym^2^
Radiation time	10.02 ± 11.36 minutes

Measurements are shown as mean ± SD unless stated otherwise.

FiO2 = fraction of inspired oxygen; iNO = inhaled nitric oxide; ppm = parts per million; PVR = pulmonary vascular resistance.

In four recruited patients at the beginning of the study, MRI guidance was not performed owing to the predictable need of a wire in a patient with a criss‐cross heart and severe valvar and subvalvar pulmonary stenosis, the patient's clinical instability (persistent pulmonary hypertensive crisis), and the artifact caused by the existing stents in the pulmonary vessels in two patients with single ventricle circulation.

The mean radiation dose and time were 0.42 ± 0.58 mGym^2^ and 10.02 ± 11.36 minutes, respectively. The total procedure time (skin in – skin out) was 219 ± 52 minutes. The median baseline pulmonary vascular resistance was 2.3 WU.m^2^ (range: 0.28–32.6). These values reduced to a median PVR of 1.9 WU.m^2^ (range: 0.63–27) in response to inhaled vasodilators.

### *Individual Adjustment of the* *pSAT* *Sequence in Clinical Cases*

**FIGURE 2:**
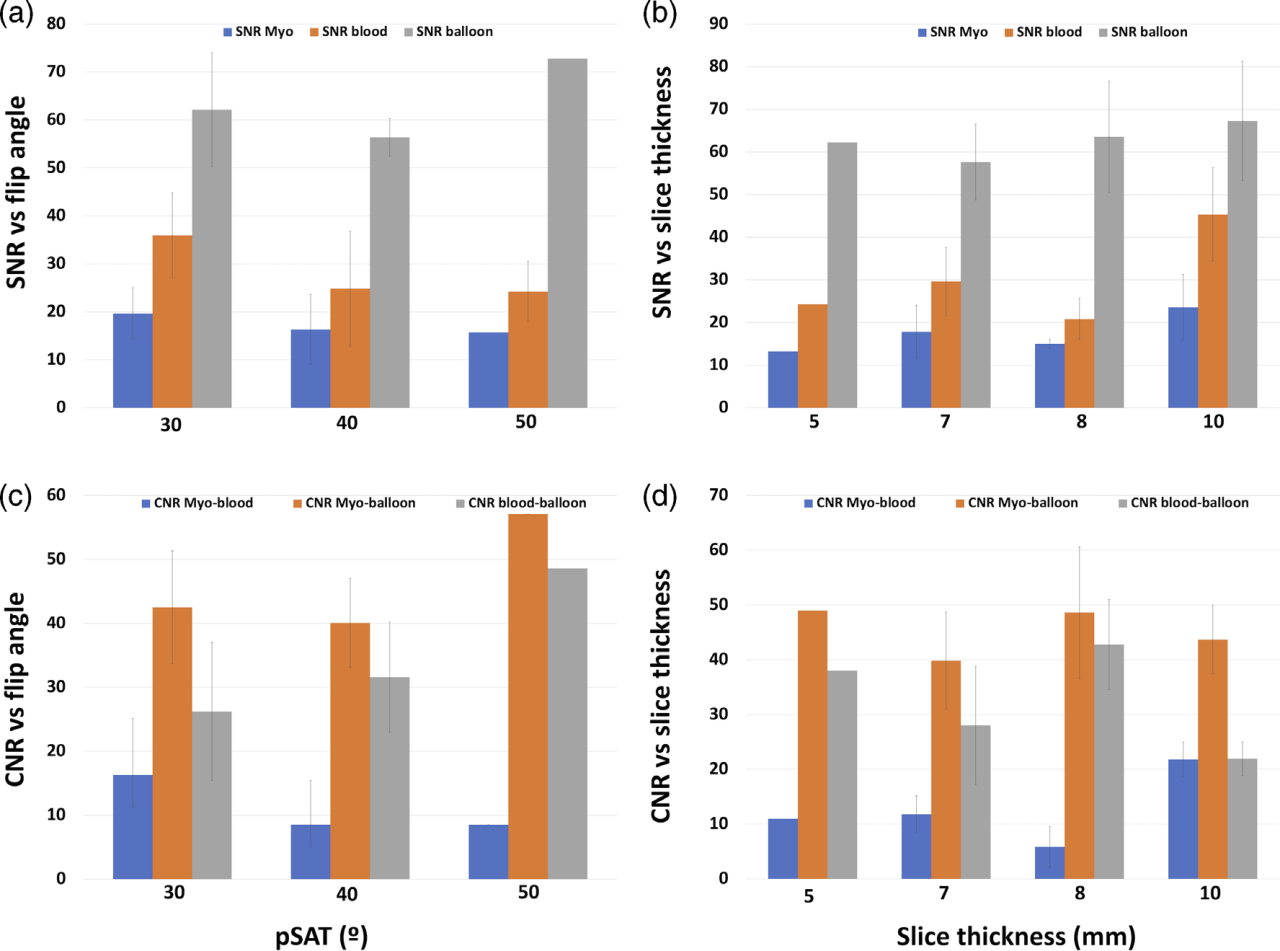
Effect of changes in pSAT angle (30°, 40°, and 50°) and slice thickness (5, 7, 8, and 10 mm) in balloon, blood, and myocardium SNR (**a,b**) and balloon/blood, balloon/myocardium and myocardium/blood CNR (**c,d**) across different patients.

The in vivo effect of changes in pSAT angle (30°, 40°, and 50°) and slice thickness (5, 7, 8, and 10 mm) in balloon, blood, and myocardium SNR and balloon/blood, balloon/myocardium and myocardium/blood CNR are summarized in Fig. 2. Only one set of pSAT angle/slice thickness was acquired for each subject; therefore, each plot represents a different patient.

**FIGURE 3:**
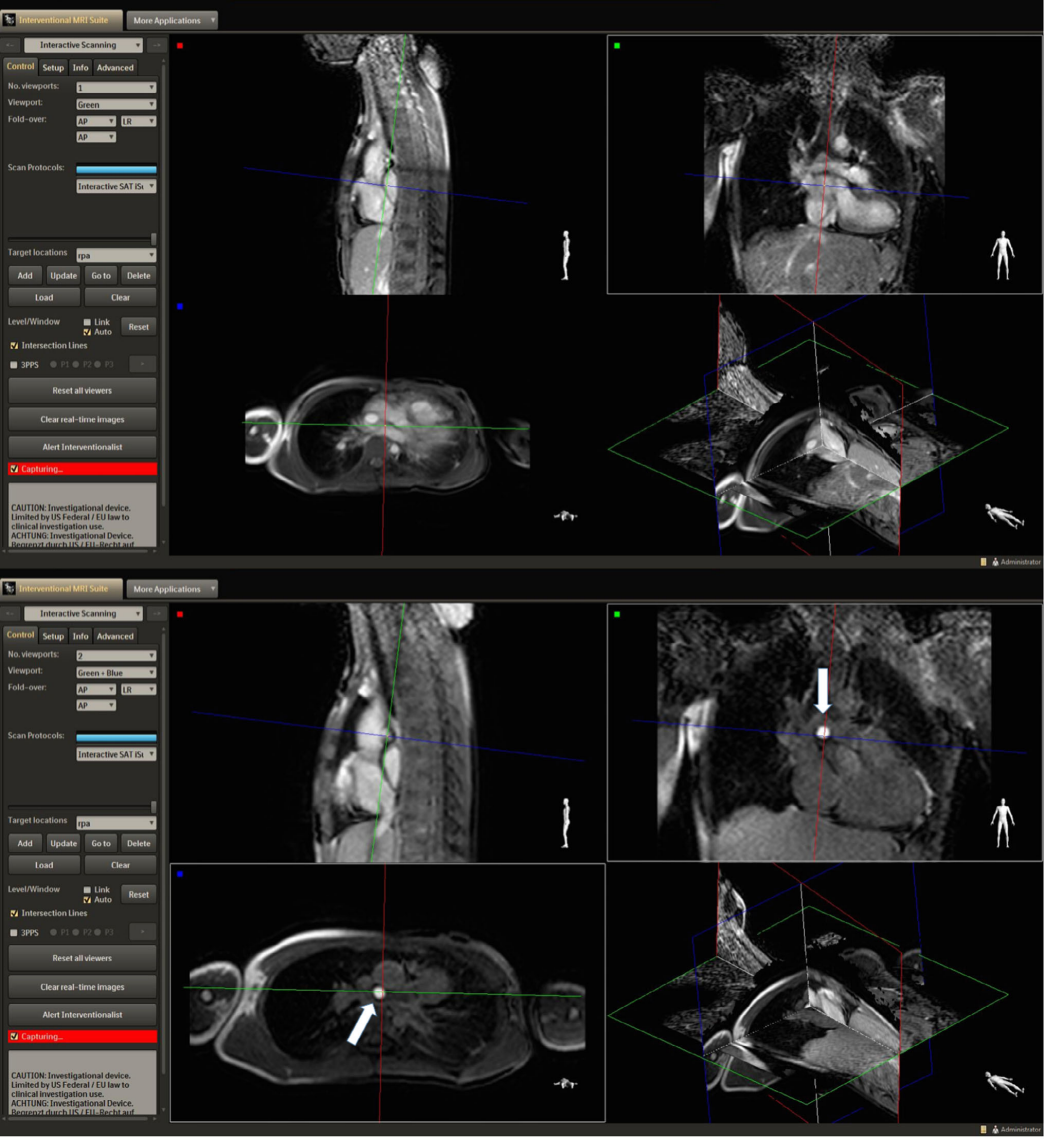
Images acquired with iSuite in a patient with Tetralogy of Fallot and LPA stent. An increase in the pSAT angle from 30° (top image) to 45° (bottom image) improved the conspicuity of the balloon‐tip by increasing the contrast between blood and balloon.

Slice thickness and pSAT angle for each patient are described in Table 1. Both features were adapted individually. In one case, the slice thickness was 5 mm. This was a small patient (weight 12 kg) with severe PA branches stenosis associated with Allagile's syndrome (minimum LPA and RPA diameter were 5 and 6 mm, respectively). For the rest of the cases, the slice thickness ranged from 7–10 mm and was adapted according to the patient's weight and cardiac structures' size. The pSAT angle to achieve partial saturation was also optimized in each case, varying from 30° to 50°. In patients with small pulmonary arteries, the turbulent flow prevented depiction of the balloon tip using low pSAT angles. This was particularly evident in a patient with Tetralogy of Fallot and LPA stent, in whom both pulmonary arteries were slender all along their course. An increase in the pSAT angle from 30° to 45° improved the visualization and allowed continuation of the study (Fig. 3, Video S1 in the Supplemental Material). A slice thickness of 20 mm and a pSAT angle of 120° was used in two cases to find the balloon during real‐time MRI catheterization. Once the balloon was located, the plane of visualization was transferred up to the balloon tip, and the slice thickness and pSAT angle converted to the one initially selected.

The volume injected in the balloon at the tip of the wedge catheter was adapted not only for each patient but according to the size of the navigated structures during the course of the case. Although initially inflated to the maximum volume recommended by the manufacturer, in numerous occasions it had to be partially deflated to cross the pulmonary valve or to catheterize the pulmonary branches.

### *Visualization During Real‐Time* *MRI‐Guided Catheterization*

The iSuite platform was used in 16 patients. In 10, the pSAT sequence was used in interactive mode in the scanner console, as the iSuite was not available. In one patient, catheter venous access was from the neck vessels with simultaneous need for a left heart catheter from a femoral arterial approach. This limited safe visualization of the iSuite screen from the head end of the MRI bore in the current set‐up of the laboratory.

Visualization of the balloon at the tip of the catheter was achieved in all the studies. During real‐time MRI catheterization, the balloon was visible throughout 57 ± 21% of the scanning time. The iSuite platform allowed for visualization in the three planes of space, with a significant improvement in the time during which the balloon was visible in relation to the interactive imaging mode on the scanner console (iSuite: 66 ± 17% vs. interactive mode: 46 ± 14%, *P* < 0.05).

Overall, the score for balloon/blood contrast and anatomical visualization was good to excellent, achieving a median of 4 (range: 2–5) and 5 (range: 2–5), respectively. High scores for both were obtained independent of the use of the pSAT sequence embedded in iSuite or as interactive mode in the scanner console (Wilcoxon test: comparison between balloon/blood contrast with iSuite or interactive mode, *P* = 0.385; anatomical visualization, *P* = 0.5).

**FIGURE 4:**
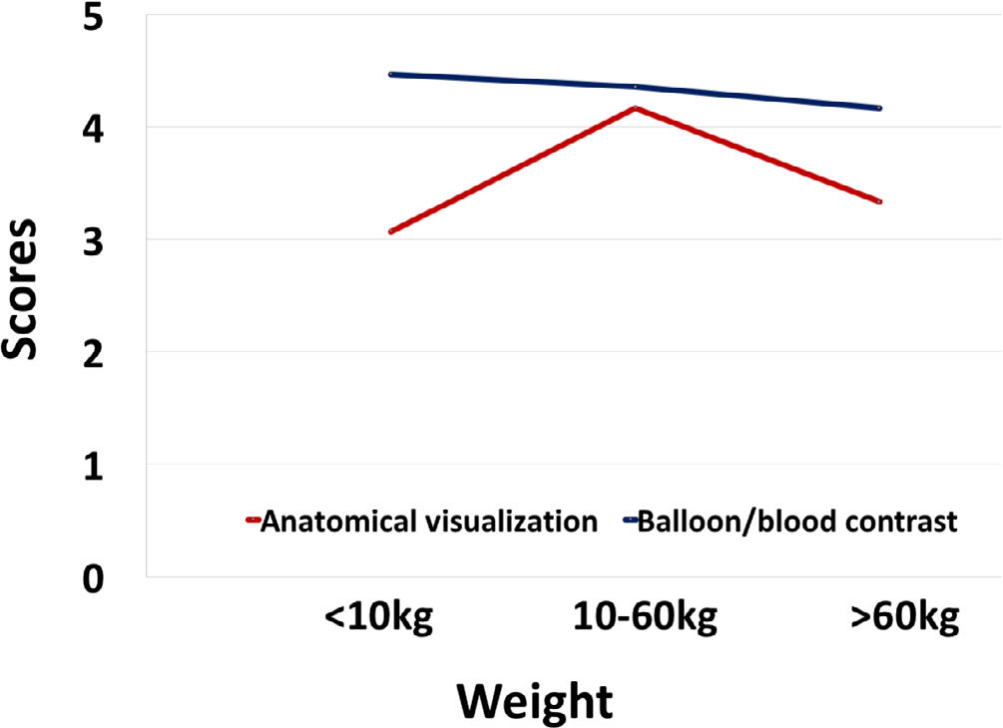
Mean scores for anatomical visualization and balloon/blood contrast scores according to patient's weight. Better scores were achieved for patients with weight between 10 and 60 kg.

Three patients received a score of 2 for anatomical visualization. Of note, all three were at the extreme side of the weight range in our population (two of them were less than 8 kg, one of them was over 95 kg). A total of 91% of scores given for anatomy depiction were ≥3; the weight range for these patients was 10–60 kg. Although no significant difference was noted, a trend to obtain higher scores was found for anatomical visualization in relation to patient's weight (*P* = 0.053). Similar values were observed when balloon/blood contrast scoring was compared among different weight groups (*P* = 0.483) (Fig. 4).

**FIGURE 5:**
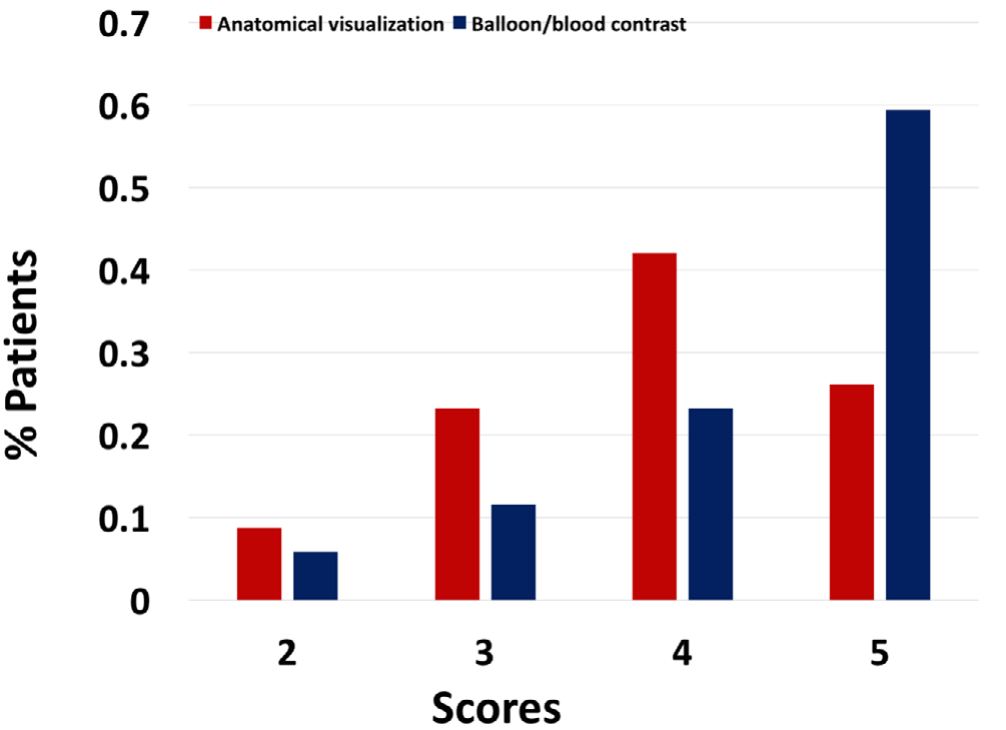
Total percentage of patients receiving a score of 2 (9 and 5%), 3 (23 and 12%), 4 (42 and 23%), and 5 (26 and 59%) for anatomical and balloon/blood contrast visualization, respectively.

Two patients in whom the MRI catheterization was guided with the pSAT sequence on interactive mode received a score of 2 for contrast. In all procedures guided by iSuite, contrast between balloon and patient's anatomy was scored ≥3, regardless of the weight of the patient. Overall, 94% of the procedures received a balloon/blood contrast score ≥3 (Fig. 5).

### *Feasibility of* *MRI‐Guided Cardiac Catheterization*

In nine patients, the procedure was completed solely under MRI (two right heart catheterization, one right and left heart catheterization, six single ventricle anatomy status post Glenn in four cases and Fontan in two).

In patients with single ventricle circulation (12 cases, five HemiFontan/Glenn and seven Fontan), the systemic venous pathway and PA branches were successfully catheterized under MRI guidance (Video S2 in the Supplemental Material, Fig. 6). However, a wire was needed to cross the fenestration to assess atrial and ventricular pressures in all Fontan cases. This was initially performed under x‐ray fluoroscopy. In three recent cases (one Fontan), an MRI‐compatible wire was used during MRI‐guided catheterization.

**FIGURE 6:**
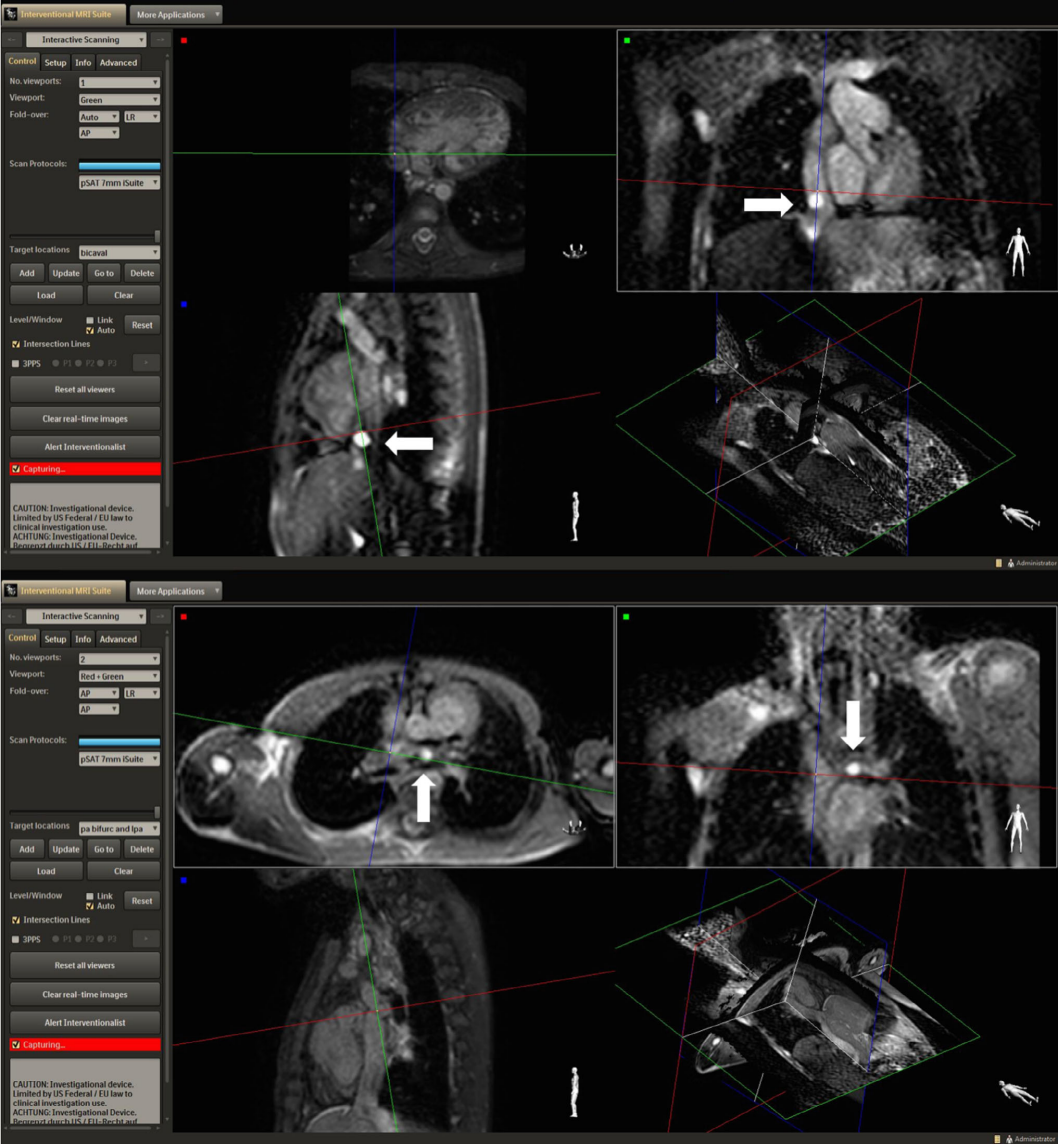
MRI‐guided catheterization of a patient with HLHS using the iSuite platform. The balloon‐tip is seen navigating the IVC (top image) and the LPA (bottom image) in a multiplanar viewer.

In 17 cases, radiographic support was employed during part of the procedure. In all of these patients, a wire was needed to cross the heart structures or reach the great vessels. In a patient with a gothic‐shaped aortic arch, a wire was needed to reach the ascending aorta from the distal arch owing to the prominent angulation of the aorta. The contrast‐filled balloon was difficult to manipulate across the tricuspid valve in one of the cases, involving a small patient (7.5 kg) in whom only a four French sheath was inserted. In patients with Tetralogy of Fallot and/or multiple discrete stenoses in the PAs and in cases with anomalous pulmonary venous drainage, a wire was needed in order to perform a subsegmental analysis of the pulmonary artery branches.

There were no MR safety complications in our study. Patient transfer between the bore and the x‐ray table when necessary was uneventful.

## Discussion

### Catheter Guidance During Cardiac Procedures

In this study we evaluated the use of our previously described novel passive tracking sequence using a partial saturation prepulse^11^ in a heterogeneous sample of patients with CHD referred for diagnostic cardiac catheterization and PVR study. Previous studies have used different software and hardware such as Interactive Front End in CHD in a relatively older population applying different passive tracking techniques from the one utilized in this study.^13^

We used commercially available balloon‐tip wedge catheters commonly used in fluoroscopy guided cardiac catheterization (balloon wedge catheter, Arrow, Teleflex, Morrisville, NC). Visualization of the balloon at the tip of the wedge catheter was achieved in all patients. Scores obtained for heart visualization showed better results for patients between 10 and 60 kg, achieving poorer scores in patients with a weight at the extreme side of the scale. The balance between conspicuity of the balloon at the tip of the catheter and clarity in visualization of the heart structures appears optimal at a pSAT angle 30°–40°, as described in our previous study.^11^ This is consistent with the selected pSAT angle for most patients. However, subtle modifications were necessary when turbulent flow was found in slender vessels such as the stenotic segments in the pulmonary arteries in some of the catheterized patients.

The need for adaptation for each case in terms of scan parameters such as slice thickness, pSAT angle, or FOV, the changes in the volume of the balloon during the case and the heterogeneity in the size of the patients and their underlying diagnosis, precludes the possibility of a more detailed statistical analysis to find out specific factors that might have an impact on the quality of the imaging when using this sequence.

Although the balloon at the tip of the catheter was not depicted during the whole scanning time, the use of the iSuite platform provided the opportunity of tracking the catheter in three planes of space and significantly increased the time during which the catheter tip was visualized. This is an important development, as it allows the operator increased control in the selection and manipulation of the imaging planes to facilitate catheter guidance.

### *Feasibility of* *MRI‐Guided Cardiac Catheterization and Procedure Safety*

Patients with complex CHD such as hypoplastic left heart syndrome (HLHS) require multiple procedures during their life. Most of the ionizing radiation they are exposed to occurs during cardiac catheterization.^18^ Out of 12 patients catheterized with functionally single ventricle circulation, six had the full diagnostic procedure performed under MRI guidance. The rest had the Fontan circulation catheterized under MRI guidance, needing radiographic fluoroscopy to cross the fenestration and measure intracardiac pressures when clinically indicated. The use of MRI to carry out at least part of the study can reduce the amount of radiation received by young patients, contributing to lessen the risk of DNA damage and developing cancer in the future.^3^, ^19^ Additionally, the hemodynamic data obtained from a combined MRI and cardiac catheter study is invaluable in CHD patients.^20^

Overall, 56% of the initially recruited patients were only partially catheterized under MRI guidance. Where small 4Fr balloon wedge catheters were utilized in small patients, they lacked the necessary torque and steerability to navigate small cardiac structures, losing their stiffness after they have been in contact with blood for a few minutes. The balloon at the tip of the wedge catheter filled in with sterile saline and gadolinium is heavier than when filled in with CO_2._ This fact may limit the use of the technique in small patients without an MRI compatible guidewire. The availability and use of the guidewire will aid in successful completion of the MRI‐guided diagnostic cardiac catheterization procedures. Recent work on metal braided catheters^21^ and metallic guidewires with low SAR sequences on 1.5T scanners^22^ may also facilitate the use of this technique.

Another challenge is the fact that the location of small patients inside the MRI scanner makes it difficult for the operator to reach the groin access site and manipulate the catheter. Although scanning off‐center would be an option for this issue, the quality of the images is suboptimal, whereas MRI catheterization requires high‐quality acquisition, especially when it comes to small and complex cardiac structures. While in other studies the majority of patients have had normally connected hearts requiring PVR studies posttransplant or before surgical correction of cardiac shunts,^12^, ^13^ our cohort of patients included severe types of CHD in very young patients (median age: 4 years, median weight: 15 kg). Although this may have caused a bias in the feasibility of completion of the study under MRI guidance, we believe our target population would benefit most from this procedure, given the need for repeated cardiac catheterization in life and the exposure to the ionizing radiation that it involves, with cumulative doses reaching high levels in the current era.^5^ This fact leads to the subsequent need for continuous development in this field.

### Limitations

The population of patients analyzed lacks uniformity in terms of size and clinical diagnosis, precluding a multivariable analysis to assess the impact of specific characteristics on the visualization of the catheter. However, it does allow for assessment of the application of this approach in a routine clinical setting of CHD.

The use of the pSAT sequence embedded in iSuite or directly in the scanner was not randomized. This, together with the fact that iSuite was used by the same operator in all cases might have created a bias and comparison of tracking times in other centers may bring different results.

Guidewires are now commercially available for use in specific MRI conditions, but a learning curve in their use will be required. This will be a subject for a future study.

## Conclusion

MRI‐guided catheterization can be carried out safely and is feasible in a wide range of young patients with CHD. This study demonstrated the robustness and reliability of the pSAT sequence for simultaneous high‐contrast visualization of the cardiovascular anatomy (tissues and blood) and the catheter balloon. However, there remains a need for additional guidewires to navigate complex anatomy, particularly in small pediatric patients with severe forms of CHD.

## Conflict of Interest

Dr. Weiss and Dr. Krueger are employees of Philips Healthcare. The other authors report no conflicts.

## Supporting information


**Video S1** MRI catheterization of a patient with TOF s/p surgical repair and LPA stent. Both pulmonary arteries are slender, the turbulent flow precludes visualization of balloon at the tip of the catheter with a pSAT angle of 30°. A change in the pSAT angle to 45° increases the contrast between the balloon and the cardiac structures.

**Video S2** Catheterization of Fontan pathway in a patient with Hypoplastic Left Heart Syndrome.
